# The Epidemiology of Maritime Patients Requiring Medical Evacuation: A Literature Review

**DOI:** 10.7759/cureus.49606

**Published:** 2023-11-28

**Authors:** Jonathan S Dillard, William Maynard, Rahul Kashyap

**Affiliations:** 1 Search and Rescue Coordinator, U.S. Coast Guard PACAREA/D11 Command Center, Alameda, USA; 2 Internal Medicine, TriStar Centennial Medical Center, HCA Healthcare, Nashville, USA; 3 Medicine, Drexel University College of Medicine, Philadelphia, USA; 4 Global Clinical Scholars Research Training, Harvard Medical School, Boston, USA; 5 Research, Global Remote Research Program, Saint Paul, USA; 6 Critical Care Medicine, Mayo Clinic, Rochester, USA; 7 Research, WellSpan Health, York, USA

**Keywords:** emergency medicine, medical transport, maritime medicine, medevac, medical evacuation

## Abstract

This literature review aims to provide an analysis of the current trends in at-sea medical evacuations (medevacs), with the objective of providing insights to decision-makers to improve patient outcomes. Sixteen sources spanning diverse research methodologies were employed for data collection. The findings point to medical disease processes outweighing trauma and psychiatric conditions as the primary justifications for medical evacuations in the international maritime community. In particular, suspected cardiovascular pathologies emerge as the most prevalent grounds for recommended evacuations, underscoring the impact of their diagnosis and treatment in maritime healthcare scenarios. Enhancing the capabilities of on-site maritime healthcare providers to obtain cardiovascular diagnostic data to facilitate shoreside interpretation is proposed to mitigate the speculations inherent to long-distance medevac decisions. Furthermore, existing research indicates that sustaining a proactive approach focusing on pre-voyage health screenings for seafarers and passenger vessel patrons holds promise in minimizing the risk of emergency evacuations resulting from the exacerbation of chronic medical conditions. This review reveals that while a limited portion of the cruise ship industry possesses established tactical medevac data on crucial aspects of patient care (such as transportation delay, pre- and mid-transport level of care, and ultimate patient outcomes), there exists a dearth of equivalent informatio­n for comparable maritime communities. This knowledge gap necessitates further exploration and research to understand and address diverse seafaring populations' unique challenges. In conclusion, this literature review holistically examines the landscape of at-sea medical evacuation statistics. By assimilating the collective knowledge gleaned from internationally sourced data, this study underscores the urgent need for continued research, comprehensive data collection, and strategic interventions to optimize patient care, enhance tactical decision-making, and ultimately shape a more resilient and responsive healthcare network for maritime communities worldwide.

## Introduction and background

The United States contains over 11.5 million vessels registered in federal database systems and a rapidly expanding U.S. maritime workforce of roughly 650,000 as of 2019 [1,2]. The cruise ship industry accommodates approximately 11 million seasonal customers in North America, while nationwide ferry transit systems facilitate over 360 million passenger miles annually [3]. Passenger and operator alike, these mariners frequently operate in remote and environmentally hostile locations isolated from essential resources easily accessible from land. With such a significant demographic of the U.S. public earning their livelihood or otherwise electing to travel via the water, a primary concern becomes providing adequate medical care to this population. Putnam (2005) has compared maritime medicine to wilderness medicine in many respects, describing frequent situations where on-hand medical equipment, accessibility of diagnostic tests, and access to appropriate specialist care are often difficult to obtain, if not entirely unavailable [4].

One of the most critical and logistically complex challenges facing maritime healthcare providers is often the most unexpected: emergency medical treatment and the potential transport of sick crewmembers to higher care onshore. Lack of onboard medical resources, limited communication capabilities, threatening environmental variables, and typically remote locations frequently create a perfect storm of logistical complexity requiring precise coordination between on-site providers, remote medical specialists, and transport asset operators to ensure a successful outcome. Medevacs are not uncommon occurrences, either. Dykes et al. found that approximately 19% of all search-and-rescue missions in the maritime environment included the need for at-sea medevacs, and Peake et al. demonstrated that the average cruise ship's physician could expect to see a life-threatening illness or injury four times per voyage with at least one requiring urgent transportation ashore [5,6]. Schemke et al. analyze the operations of search-and-rescue providers, utilizing triage severity scores to demonstrate that the disease and injury acuity for maritime missions were much higher than those reported in an average city's land-based emergency medical services [7].

These high-gain and frequently high-risk operations involving the evacuation of critically ill patients in the maritime environment demand a background of clinically geared data to inform tactical decision-makers. Streamlining the safety of at-sea medical transports, improving patient outcomes, and promoting a strategically superior medevac culture is contingent upon research sensitive to the trends in medical needs specific to the seagoing community. To identify strengths and potential data gaps relevant to the above topic, we reviewed research surveying patient epidemiology in various maritime settings requiring emergency interventions or medical evacuation to a higher level of shoreside care. This review could supply maritime healthcare providers, both shipboard and land-based, with valuable data to guide future emergency response efforts and preventative medical measures to safeguard life at sea.

## Review

Methods

We selected sources from various research accessible via an American Public University System subscription. These include the Trefly Library, Proquest.org, and keyword searches via Google Scholar. All material was accessed between December 8, 2021, and January 10, 2022, with data collected using the search terms "emergency," "medical," "maritime," and "medevac" from the above databases. Included research contains results from data extending as far back as 1935 to the present decade. Additionally, the references from each utilized research source were assessed for sources relevant to this review. Rejected sources included medical literature and research into emergency preparedness and transportation logistics lacking sufficient maritime relevance. In addition, news articles and miscellaneous publications featuring specific case scenarios from maritime-based medical emergencies were excluded, together with sources offering no quantitative data to inform our results. Finally, statistics offering no distinction between medically routine/critical cases (let alone emergency evacuation) were excluded for ultimate inconsequence to our review objective. Studies were retained based on their relevance to a quantitatively informed characterization of at-sea emergency medicine and specifically discussion concerning the maritime community's reliance on transport services for a higher level of care in emergent medical situations.

Reviewed Literature

**Figure 1:**
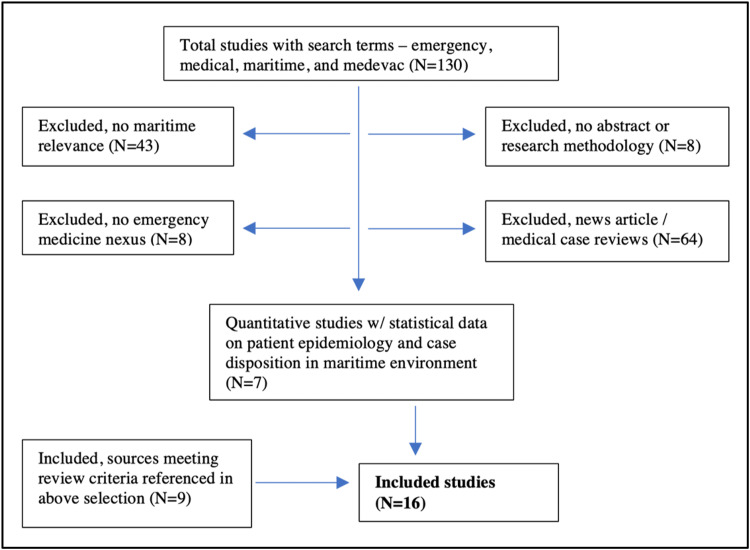
Research inclusion criteria

One hundred thirty sources were evaluated for relevance to our literature review objectives utilizing the above search methodology. Sixteen were ultimately utilized to form the core of the data driving our discussion, and 123 were excluded for reasons detailed in Figure 1 below.

Though existing literature discusses the technicalities of shipboard medical care (including critical-care situations) at length, there is less analysis of shipboard care coordination necessitating outside resources, such as medical counsel or evacuation to a higher level of care (termed MEDICO and MEDEVAC, respectively, by search-and-rescue agencies including the U.S. Coast Guard). Multiple articles analyze specific medical complaints and resulting treatments within maritime settings such as cruise ships, some of which did indeed escalate to medevac operations. Others offer a more comprehensive analysis of medical assistance requests for a specific region, irrespective of platform type. Other sources are didactic rather than experimentally based, detailing the characteristics of particular maritime rescue organizations or using a data-driven approach to justify minimal qualification standards for onboard medical providers. Additional research yields insights into the role of advanced communications technology, such as secure radio or virtual telemedicine capabilities, and evaluates their feasibility in the emergency maritime medical setting. Finally, an assortment of case reviews discusses specific shipboard medical scenarios to provide insights into acute injuries either likely to occur in or be complicated by their shipboard environment.

Research meeting qualification for inclusion in this review consisted of quantitative data relevant to the at-sea medevac decision and can be divided into three subgroups. The first is research that surveys the specific epidemiology of a particular maritime sub-group, typically intending to introduce preventive measures ranging from elevated equipment safety standards to additional medical training required for shipboard providers. The next body of research focuses on the relationship of shipboard health providers with outside emergency medical specialists via various communication methods, with the primary goal of optimizing the capabilities of virtual medical services offered to mariners. The third collection of sources covered in this review analyzes the operational endeavors of various medevac organizations dispatched to emergency medical scenarios warranting higher levels of care. Metadata regarding medevacs from U.S. military platforms to include Coast Guard or Navy vessels was not available to an extent adequate for this review. No patient health data containing personally identifiable information (PII) were evaluated during this review.

Results

Maritime Community Epidemiology

This first collection of research evaluates the overall epidemiology of various maritime communities-particularly passenger vessels. Though this research was not aimed at identifying trends in evacuation statistics, it includes all medical cases or traumatic injuries requiring any patient-provider interaction, with at least a minimum nexus to emergency evacuation (or situations warranting critical care intervention) to merit the data's inclusion in our review. Excluded were multiple studies solely evaluating traumatic injuries or fatal injuries onboard their chosen subset of vessels. The obtained data are included in Table 1 below.

**Table 1:** Maritime community epidemiology

SN.	Author (year)	Time Span	Geolocation	Vessel type	Cases Onboard	Case Disposition
1	Peake et al. (1993) [6]	1 year	Americas	Cruise ships (~1,143 avg passengers)	N = 7,147 Medical – 69.3% Trauma – 18.2% Other – 12.5 % Critical – 11%	Remote consultation w/ physician – 1.3 % Ashore referral – 8.2% Evacuation – 2.75%
2	Dahl, E. (2005) [7]	106 days	Los Angeles to New York City	Cruise ships (1,079 avg passengers)	1,467 patient contacts	Evacuated – 0% Referred ashore – 7% Hospitalized in port – 8%
3	DiGiovanna et al. (1992) [8]	2-7 months (various cruises)	Caribbean	Cruise ships	N = 1,574 Medical – 88% Trauma – 12% Non-Critical – 97% Critical – 3%	Not provided

Epidemiology of Remote Medical Advice (MEDICO)

The second subset of data meeting inclusion criteria lacks the broader maritime community health statistics obtained in the previous section. It only includes cases communicated to remote healthcare partners where onboard healthcare providers had already recognized their need for guidance from a higher level of care onshore. This trend is reflected in reported patient-provider contacts' higher average acuity level. Most research was collected and analyzed by various worldwide telemedical communication hubs specializing in offering medical counsel and logistical coordination of medevacs when necessary. Data are detailed in Table 2 below.

**Table 2:** Medevac correlation to onshore MEDICO statistics

SN.	Author	Time Span	Facility	Vessel Type	Calls	Incident Type	Case Disposition
1	Mitchelson et al. (2008)[9]	12 months	Maritime and Coastguard Agency (United Kingdom)	Fishing – 38% Oil support – 37% Leisure – 21% Oil rig – 20% Other – 32%	186	Trauma – 53% Medical – 47%	Evacuated - 85% of fishing vessels, 73% of other vessels, 73% of trauma cases, 97% of medical cases
2	Mahdi, S. and Amenta, F. (2016)[10]	10 years	Centro Internazional Radio Medico (Rome, Italy)	Not provided	27,473	Critical – 13.5% non-Critical – 86.5%	Not provided
3	Lateef, F. and Anantharaman, V. (2002)[11]	21 years	Singapore General Hospital	Not provided	2,320	Gastrointestinal – 25.1 % Musculoskeletal, trauma, burns – 12.9% Fever – 11.5% Ureteric/renal colic – 5.6% Cardiovascular – 5.1% Other – 39.8%	Evacuated – 12.3% Vessel diversion – 27.4% Aid at next port – 37.8% Treated onboard – 60.3%
4	Szafranrowolska, R. and Wolyniec, W. (2020)[12]	6 years	Telemedical Maritime Assistance Service (Poland)	Not provided	225	Infections – 27% Trauma – 15% Abdominal – 13% Cardiovascular – 12% Other – 67%	Evacuation – 49 (20%) Cardiovascular – 26% Trauma – 24% Abdominal – 10% Miscellaneous – 40%
5	McKay, M. (2007)^[13]^	2 years	Various	U.S. flagged	120	Medical – 84% Trauma – 14% Psychiatric – 2%	Not provided
6	Charalampos et al. (2017)[14]	1 year	Med Solutions International	Primarily container ships and tankers	551	Cardiovascular – 8% Other – 92%	Of cardiovascular-related cases: Evacuated – 9% Referred ashore – 72% Other – 19%
7	Thibodaux et al. (2014)[15]	5 years	n/a	U.S. Gulf Coast oil rigs	8019	N = 102	Evacuated - 5% Nonoccupational - 77% Occupational - 23% Cardiovascular - 45%
8	Çakir, Erkan and Arslan, Ömer (2018)[16]	4 years	Telemedical Maritime Assistance Service (Turkey)	International waters – 78% Turkish coastal waters – 22%	5080	Mariner injury & poisoning – 44.4% Fisherman & Passengers Cardiovascular – 12.6% Other medical conditions – 7.7%	Mariners evacuated – 10.4% Fisherman & passengers evacuated – 82.3%
9	Westlund, et al. (2016)[17]	6 years	Telemedical Maritime Assistance Service (Sweden)	Commercial merchant ships and high-passenger ferries	Mariners - 1095 Passengers - 651	Mariner general accidents – 20% Passengers Cardiovascular – 16.5%	Mariners evacuated – 15% Passengers evacuated – 39%

Research Gathered by Medevac Partners

The third group of sources utilized in this literature review exclusively involves patients involved in medevac operations. Understandably, information included in previously mentioned sources, such as broad seafarer epidemiology or correspondence that failed to merit evacuation, is excluded in this section. Data are included below in Table 3.

**Table 3:** Medevac statistics from transport providers

SN.	Author	Time span	Vessel type	Location	Evacuations	Medevac Asset	Transport details
1	Prina et al. (2001) [18]	9 months	Cruise ships	Bahamas, Caribbean, and Central America	N = 104	Aeromedical transport – 100%	Physician accompanied - 79.8% Nurse/Paramedic – 20.2% Mortality during transport - 2.9%
2	Dykes et al. (2011) [5]	14.5 months	Fishing – 28% Military – 20% Dive boats – 12% Passenger vessels – 11% Oil rigs – 10%	United Kingdom	N = 172 Medical – 53% Trauma – 47%	Helicopter – 100% Landed onboard – 13% Winched onboard – 87%	Royal Air Force/Navy helicopters with paramedic onboard
3	Holt et al. (2017) [19]	3 years	Ferries (2,600 passenger)	Oslo, Norway to Kiel, Germany	N = 169 Cardiovascular – 40% Stroke – 9.4% Fractures – 9.4% Abdominal – 8.2 % Respiratory – 7.1 % Obstetrics/Gynecology – 5.9% Other – 20%	Helicopter – 50.3% Boat – 2.4% Pierside – 47.3%	Not provided
4	Boudsocq, et al. (2019) [20]	13 years	Not provided	Marseille, France	N = 2,375	Rescue boat – 100%	Marseille Fire Brigade physician accompanied – 8%

Discussion

Epidemiology of Maritime Evacuations

Research obtained during this review points to medical complaints and illnesses as a more significant burden to the maritime healthcare provider than other injuries often associated with the elevated lifestyle risks of mariners, such as trauma or drowning. Dahl identified medical complaints the onboard healthcare team treated as outnumbering traumatic injuries more than 14:1 onboard surveyed cruise ships [21]. Peake et al. likewise identified that over 69% of reported passenger maladies onboard cruise ships were directly tied to medical complaints, 11% of which required critical intervention [6]. Although this is an expected trend for the typically elderly passengers with preexisting chronic health conditions found onboard cruise ships, this trend holds for other maritime communities. Surveying calls made to onshore medical providers, McKay makes the case that medical cases dominated at 84% on a wide selection of U.S. flagged ships, and Dykes et al. found that a plurality (28%) of all maritime medical evacuations performed in the U.K. were from fishing vessels [13,5].

Medical concerns remain more prominent than maritime injuries when evaluating evacuation statistics. In research conducted by Mitchelson et al., an astonishing 97% of all medevacs performed over one year were medically related, with traumas far more likely to be successfully managed by onboard providers (of note, 85% of these calls were received from the fishing vessel fleet rather than passenger vessels) [9]. Per Thibodaux et al., preexisting medical conditions were far more likely to result in a medevac than any injuries exacerbated by the underway workplace environment, with nonoccupational medical conditions accounting for 70% of medevacs from Gulf Coast oil platforms compared to those considered "occupational" [15]. While isolated sources such as Boudsocq et al. surveying medevac cases just offshore municipal areas such as Marseilles, France, list typical traumatic injuries such as boating collisions, swimming, diving, or drowning as more common than medical concerns, the trend holds that medical complications, often preexisting, dominate the landscape of pre-hospital emergency care in the offshore maritime environment [20].

Various sources propose remedies to address this correlation of medical, often chronic, health problems with the need for medical support up to and including the possibility of at-sea medical evacuation. Various maritime employers widely emphasize and implement pre-screening of passengers and crew for preexisting chronic health conditions. The medical training of onboard health care providers on passenger vessels is recommended to be as generalist as possible, with specialization in family or emergency medicine recommended to adequately address the wide variety of medical complaints presented in onboard clinics [18]. Paramedic-trained providers are used effectively on high-passenger ferries in Norway to triage patients for rapid transport off-vessel if needed [19]. Given the often-limited medical training offered to merchant ship medical officers often burdened by demanding collateral duties, McKay recommends specific training in basic dermatological and dental medicine to eliminate a notable portion of preventable underway medical situations that tend towards escalation [13].

Maritime Cardiovascular Cases

The wide range of cardiovascular medical conditions that quickly surpass the level of care on board the vast majority of vessels frequently necessitate urgent medical correspondence and constitute a sizable proportion of time-sensitive medical evacuations. Szafranrowolska and Wolyniec note that cardiovascular complaints were the third most common cause of communication with outside medical providers and the most common reason (26%) for a recommended medical evacuation [12]. Other sources agree, with Holt et al. noting that 40% of all evacuations onboard the Oslo-Kiel ferry are cardiovascular, comprising the most considerable portion of patient transports [19]. Similar statistics were obtained from a survey of evacuations performed for U.S. Gulf Coast oil rigs from 2008 to 2012, with 45% reported as cardiovascular [15]. Prina et al. find that a plurality of 27.8% of all medical evacuations from U.S. cruise ships from 1999-2000 were cardiovascular to include ailments such as myocardial infarction, angina pectoris, arrhythmias, and cardiac arrest (a total of 14% of which died either during transport or post-hospitalization) [18]. Additionally, Çakir and Ömer find that a plurality (12.6%) of medical calls for fishermen and other non-professional seafarers are cardiovascular, with 82.3% of all cases eventually being recommended for medevac ashore [16]. Similar research by Westlund et al. concurs, establishing medical concerns, particularly cardiovascular complaints, as a primary medevac justification for seagoing passengers with evacuation rates outweighing those of other commercial mariners 39% to 15%, respectively [17].

The commonality of cardiovascular complaints at sea is intertwined with the ability to effectively diagnose the severity of acute cardiac events in a resource-limited environment. On remote communications-limited vessel platforms without the ability to obtain or transmit vital diagnostic information, the prevalence of cardiac "themed" complaints could result in a transport decision being made when not medically necessary. In researching the outcomes of cardiac cases reported from major international shipping companies in 2016, Apostolatos et al. conclude that only 9% of all evacuated cases required urgent medical transportation for a successful patient outcome, while other cases could have been satisfactorily referred ashore for specialist evaluation at the vessel's next port of call [14]. These data highlight some of the challenges facing diagnosticians in the maritime environment as opposed to, for example, a rural land-based setting where diagnostic tools (e.g., ECG) could be utilized on-scene to determine transport priority. Given the lack of concrete medical information for many patients, erring on the side of ultimately unnecessary medevac operations can incur additional concerns such as cost and, more importantly, undue risk to operators and patients alike. Charalampos et al. note that the psychological stress associated with an at-sea medevac (often including hoisting from a moving deck to an airborne helicopter) could be involved in symptom exacerbation and strongly recommend that merchant ships include basic training in ECG acquisition and transmission as well as outfitting vessels with the ability to acquire basic blood labs such as troponin levels to inform medevac decisions for cardiac patients [14]. Managing the mariner or shipboard passenger with acute-onset cardiovascular symptoms continues to constitute the largest segment of at-sea medevacs and, potentially, the unnecessary utilization of medevac assets in a particularly resource-scarce environment.

Maritime Medevac Operations

A final category of data discussed in this review contains details pertinent to the emergency medical transport itself, if performed. The first and most apparent differential is that of the asset utilized for medevacs. In the U.S. Coast Guard's data systems, small boat transportation of individuals with injuries due to collisions and other boating accidents are often not strictly categorized as medevacs, which could underrepresent usage of this asset in addition to patient epidemiology statistics. In the vast majority of other reviewed research, the utilization of surface vessels for transport was almost non-existent save the work of local first responders in Marseilles, France in the context of a municipal small-boat rescue organization [20]. In other situations, vessel deviation or pier-side transport at the next available port of call was adequate given the medical situation, representing 47.3% of evacuations by high-passenger ferries between Norway and Germany [19]. Since the operating area of medevac recipients often lies well beyond the range of surface assets, helicopter-facilitated medevacs were by far the predominate asset utilized for off-vessel transportation in virtually all other reviewed research, with airborne winching of the patient typically employed over shipboard landing and embarkation due to weather concerns or vessel construct limitations [5].

Additional considerations include actual patient transport, a vital concern due to critically ill patient outcomes being closely related to prompt arrival at definitive medical care. Alone of all research analyzed in this project, Prina et al. include average delay of arrival time frames for each category of illness evacuated from cruise ships, with ischemic and hemorrhagic strokes representing the most prolonged delay until reaching hospital care [18]. Of note, these statistics are gathered from cruise ships located in the Caribbean at the time of medevac, with transports often prolonged to deliver patients to optimal levels of care in the U.S. instead of less-capable emergency departments on nearby islands [18].

Another concern is that of delineating the level of care provided mid-transport. Boudsocq et al. calculate the number of missions a physician was requested to accompany local small boats on regional medevac missions at only 8% [20]. Prina et al. correlate cruise vessel evacuation outcomes with the level of care received during each flight to determine an ultimate diagnostic accuracy of 90.4%, with minimal cases requiring a higher level of care than was provided during transport [18]. This retrospective analysis is not surprising, as medevac decision-makers in the U.S. Coast Guard will sometimes deny transportation requests and leave the patient onboard rather than risk a fatality en route if the level of care provided by the medevac asset will substantially decrease. Other sources remain silent on this critical metric.

Knowledge Gap

The review of existing research above engages multiple questions relevant to the remote coordination of shipboard emergency medical care, though knowledge gaps certainly exist. Population epidemiology and evacuation statistics are well-researched for the cruise line and passenger vessel industries in varying geographical locations but sparingly for other communities such as the commercial fishing fleet. Studies specific to fishing vessels aimed to implement additional safety measures onboard and thus only included data such as work-related mortalities or traumatic injuries [22,23]. Though telemedical centers maintain databases covering broad swathes of the medical community regardless of vessel type, a study into specific maritime demographics, such as fishing vessels, would be ideal to cater to the healthcare needs of maritime workers comprising significant economic populations.

Of additional concern, the risk-inherent step of the medical decision-making process, the at-sea transfer of a patient to another asset for emergent transport, is primarily featured as a medical case disposition in existing research rather than a subject for devoted inquiry in most sources. There exists little statistically grounded research delving specifically into the multifaceted logistical complications (such as time delay, reduction of care in transit, or threat of a failed hoist operation) that ensue when involvement from ashore medical facilities extends beyond medical advice and to the physical evacuation of an ill patient to a higher level of care. In these circumstances, a comprehensive and data-informed understanding of an enormous set of risk variables and their implications could immediately impact tactical decision-making in future operations. 

Very little research on patient epidemiology also contains data on, for instance, the total number of hours sacrificed to the medevac process. Beyond Prina et al.'s discussion of transport time (conveniently broken down by pathology classification) for cruise ship patients in the Caribbean, similar data sets for other maritime communities are not available. In addition, reviewed sources overlooked the impact of transportation factors such as weather or fuel capacity on medevac operations. These are concerning gaps as the time taken to medevac a patient and the ability to safely do so successfully is a critical decision in determining whether or not a patient will benefit from being physically transferred to a higher level of care.

Another research gap relevant to medevac decision-makers is determining where suitably trained healthcare providers are needed to provide maximum flexibility to underway medical evacuation options. This statistic is of vital importance as U.S. Coast Guard helicopters providing medical transports are typically staffed with Aviation Survival Technicians or other crewmembers whose skills are specialized in "challenging aquatic helicopter rescue," with medical skills extending to basic life support interventions and "not necessarily the care of complex cardiac patient, or someone on a ventilator" for instance [21]. Beyond Prina et al.'s inclusion of this information in their geographically limited research of cruise ship passengers, there exists scarce data specific to maritime patient outcomes to inform the appropriate level of providers onboard medevac assets. The physiological stress and unique tactical difficulties of maritime medevacs, often involving airborne hoisting of patients and potential accompanying healthcare providers, necessitates additional research similar to that conducted for land-based flight medevac services.

Finally, in-depth cost analysis of medevacs could be improved for most maritime situations. Thibodaux et al. include this detail in their discussion of oil platform medevacs, which contract their evacuation services from commercial providers at an average of $54,000 per flight [15]. Other vessels in the United States typically utilize the U.S. Coast Guard for medevac services, which are free of charge to the consumer and paid by the government. Nonetheless, determining the suitability of a medevac is frequently (and unfortunately) tied to cost-relevant decisions such as diverting a scheduled cruise ship or merchant vessel to the nearest port-of-call or location optimal for a medevac rendezvous. A patient's possession of travel insurance with which to fund a medevac can also impact the course of action to which a vessel will more eagerly consent. This conversation with budget-cognizant vessel masters can complicate medevac decision-making and stands to benefit from a comprehensive cost-benefit analysis of evacuations involving vessel diversion from charted voyage plans to effect ideal patient outcomes.

Recommendations

This review supports prospective policy implementation in line with many source recommendations. Firstly, it underscores the importance of establishing acceptable regulatory requirements for mariner health standards before individuals embark on various maritime platforms. Collecting prior medical history can also significantly assist healthcare providers in making informed decisions regarding medevac eligibility. Secondly, considering the common occurrence of cardiovascular complaints that often go undiagnosed in the maritime environment, this review advocates for the widespread adoption of shipboard telemedical devices, including ECG, to streamline remote diagnosis.

Future inquiry could involve conducting dedicated studies to assess the overall epidemiology and medevac patient outcomes within lesser-researched maritime communities, such as U.S. fishing vessels, to provide specific tactical insights to shoreside decision-makers. Additionally, a comprehensive review of U.S. Coast Guard medical transportation data to include risk assessment, delay in care, level of care administered during transportation, and correlations with patient outcomes could provide valuable data absent from this review.

## Conclusions

We conducted a literature review to understand factors of patient epidemiology relevant to at-sea evacuation. From 16 relevant sources, we found that medical situations necessitating medevacs for patients onboard passenger vessels such as ferries and cruise ships are well-researched but that records specific to other segments of the maritime population such as recreational vessels, fishing fleets, or oil rigs are obtained only from cases referred to outside medical advice or eventual evacuation and do not cater to the entirety of medical realities experienced by these communities-data that could help determine the adequate level of training for onboard health care providers, for instance. Details relevant to patient outcomes, such as delay to definitive medical care, level of care in transit, and overall quality improvement of diagnostic accuracy and medical interventions en route, exist for the cruise ship industry but, again, are lacking in other seagoing arenas. Overarching strategic questions such as a cost-benefit analysis of alternatives to government-provided aeromedical evacuation services (such as contracted flight services) while examining patient outcomes specific to diagnosed condition is absent from existing research. The majority of the literature surveyed in this review strongly recommends more effective pre-employment or voyage health screening and improved communications technology with onshore medical providers to include the acquisition and transmission of basic medical vitals (blood pressure, remote auscultative capabilities, lab work, and ECG) and additional training of ship medical officers. These research gaps compromise the ability of government organizations and private companies providing at-sea medevac services to optimize their services for maximal benefit to the seagoing mariner and the vital industries they support.
